# Impact of major awards on the subsequent work of their recipients

**DOI:** 10.1098/rsos.230549

**Published:** 2023-08-09

**Authors:** Andrew Nepomuceno, Hilary Bayer, John P. A. Ioannidis

**Affiliations:** Meta-Research Innovation Center at Stanford (METRICS) (AN, HB, JPAI) and Departments of Epidemiology and Population Health (AN, JPAI) and of Medicine (JPAI), Stanford University, Stanford, 94305-6104, USA

**Keywords:** awards, bibliometrics, research incentives

## Abstract

To characterize the impact of major research awards on recipients' subsequent work, we studied Nobel Prize winners in Chemistry, Physiology or Medicine, and Physics and MacArthur Fellows working in scientific fields. Using a case-crossover design, we compared scientists’ citations, publications and citations-per-publication from work published in a 3-year pre-award period to their work published in a 3-year post-award period. Nobel Laureates and MacArthur Fellows received fewer citations for post- than for pre-award work. This was driven mostly by Nobel Laureates. Median decrease was 80.5 citations among Nobel Laureates (*p* = 0.004) and 2 among MacArthur Fellows (*p* = 0.857). Mid-career (42–57 years) and senior (greater than 57 years) researchers tended to earn fewer citations for post-award work. Early career researchers (less than 42 years, typically MacArthur Fellows) tended to earn more, but the difference was non-significant. MacArthur Fellows (*p* = 0.001) but not Nobel Laureates (*p* = 0.180) had significantly more post-award publications. Both populations had significantly fewer post-award citations per paper (*p* = 0.043 for Nobel Laureates, 0.005 for MacArthur Fellows, and 0.0004 for combined population). If major research awards indeed fail to increase (and even decrease) recipients' impact, one may need to reassess the purposes, criteria, and impacts of awards to improve the scientific enterprise.

## 1. Introduction

Research awards often include in their statements of purpose intent to reward, motivate and support scientists in contributing even more important work in the future. The emphasis on stimulating future work is more prominent for awards given to young researchers, but it applies even to those given to senior, long-established investigators.

The Nobel prize, arguably the most prestigious award, ‘was designed both … as reward for past major contributions to science and as incentive for future ones’ [1], and it aimed to ‘help men of promise develop further’ [2]. The MacArthur Foundation fellowship program (aka ‘Genius Grant’), arguably the most prestigious award covering all sciences and all other types of creative enterprise and focusing mostly on young overachievers, is described as ‘an investment in a person's originality, insight, and potential’ intended ‘to enable recipients to exercise their own creative instincts for the benefit of human society’. MacArthur conditions its awards upon ‘promise for important future advances' and ‘potential for the Fellowship to facilitate subsequent creative work’ [3].

However, how positively do Nobel Prizes, MacArthur Fellowships and other prestigious accolades—intended by donors to boost subsequent achievement—influence scientists' contributions? One possible means to assess this is to analyze winners' citation counts for their work preceding and work following an award. Despite its flaws, the citation metric is commonly used by the scientific community as a proxy for impact [4,5].

Others have studied scientific prize winners with an eye to understanding how awards affect subsequent contributions. In 1967, sociologist Harriet Zuckerman reported that on average, Nobel Laureates publish fewer papers after being named, and that reduction in output is more pronounced for those for whom the award entails larger gains in social status [6]. Researchers have yet to discover whether the same pattern exists in more recent laureates. Borjas and Doran [7] used controls—comparing winners to ‘similarly brilliant contenders'—to show that after receiving the Fields Medal in mathematics, recipients decrease their publication rate substantially [7]. By contrast, Chan *et al*. [8], who also compared awardees with controls, found that recipients of the Clark Medal and Econometric Society Fellowship increase both productivity (publication count) and impact (citation count). More recently, Li *et al.* demonstrated that Nobel Laureates experience a dip in citations—but not publications—immediately after winning the prize, but recover to pre-award citation rates four years later. They also corroborated Zuckerman's finding that after receiving a Nobel, laureates make greater changes to their research careers than do their peers [9].

We've aimed to contribute some evidence on the impact of awards on the subsequent work of their recipients by investigating Nobel Laureates' and MacArthur Fellows’ pre- and post-award contributions as measured by citation counts and citations per paper.

## 2. Methods

### 2.1. Study population

We studied Nobel Prize winners in chemistry, medicine and physics. We also studied MacArthur fellows who worked in a field that might be best described as science (rather than other areas of creativity) and had some minimum impact in the scientific literature.

We chose these populations in order to include scientists in the beginning and middle of their careers (as is typical of MacArthur Fellows), as well as those nearer the end (as is typical of Nobel Laureates). The relationship between age and observed scientific creativity is well-documented and varies with changes in field-specific training patterns and prevalence of theoretical contributions [10]. Subjects received at least one of these awards between 2004 and 2013.

**Figure A1.**
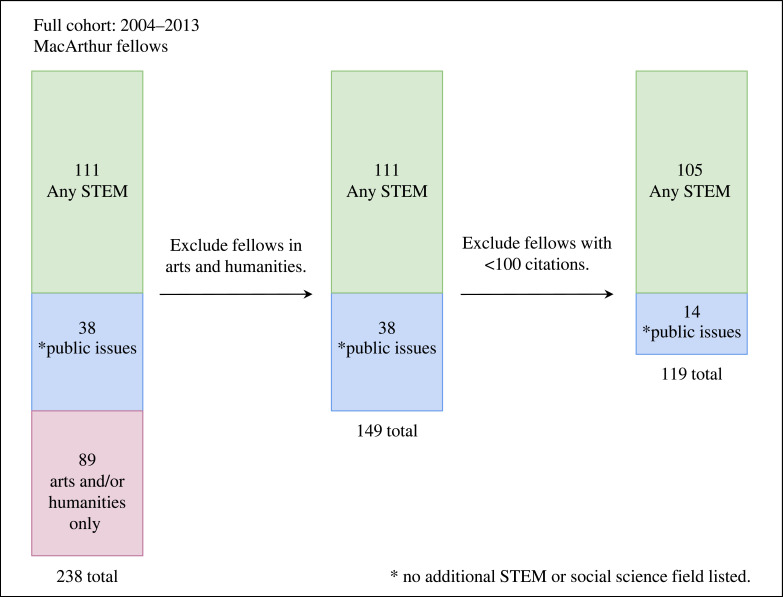
Exclusion of MacArthur Fellows by field.

We included all 72 Nobel Laureates and 119 of 238 MacArthur Fellows (see Appendix). We excluded 89 MacArthur Fellows because they published in fields that the MacArthur Foundation classified as Arts and Humanities. We excluded 29 Fellows who had earned too few (less than 100) total career citations to allow meaningful pre- and post-award comparisons. We further excluded one fellow who had no official Scopus profile. Appendix table A1 lists all fields into which the MacArthur Foundation classifies Fellows' work, and Appendix figure A1 depicts how MacArthur Fellows were included or excluded.

### 2.2. Study design

In this matched case-control study, we compared each scientist's pre- and post-award citations. Thus, each person serves as her or his own control. This design is, therefore, a case-crossover study.

### 2.3. Data collection

For every scientist who met selection criteria, we recorded age at time of award; field of study; and pre- and post-award citation counts, publication counts and citations per publication. We defined pre-award papers as those published during the two years immediately before and during the year of an award, and post-award papers as those published during the second through fourth years after the year of an award. Thus, both the pre- and post-award periods are only 3 years.

For pre-award and post-award papers, we counted citations, publications and citations per publication accumulated during equal durations. For example, if a scientist won an award in 2004, the pre-award citation count includes citations received between 2002 and 2015 on papers published during 2002–2004, and the post-award citation count includes citations received between 2006 and 2019 on papers published during 2006–2008. We exclude papers published in 2005, because they may represent work done either before or after winning the award. Citations are counted until the end of 2019 for the post-award work and until the end of 2015 for the pre-award work. This means that for each awardee, 4–13 years of citations are counted depending on when the award was given, but for all of them citations for the pre-award and post-award work are counted over the same duration of time.

We identified MacArthur Fellows and Nobel Laureates on macfound.org [11] and nobelprize.org [12], and harvested citation counts from Scopus. Citation data for all papers of all study subjects were scraped from Scopus using the Python-based API-Wrapper Pybliometrics [13]. Scraped data were validated against manually collected data for a random sample of authors. Data and analysis code are available at Dryad [14].

### 2.4. Statistical analysis

Our original primary metric of interest was citation counts, but we added publication counts and citations per publication as additional metrics, after feedback from peer-reviewers. For each scientist, we computed citation, publication, and citations per publication difference scores by subtracting pre-award counts from post-award counts. We calculated median difference scores and tested with a Wilcoxon signed-rank test the null hypothesis that pre- and post-award work impact does not differ.

To determine whether research awards have heterogeneous effects on scientists in different career stages, we stratified in an exploratory analysis the evaluated researchers by age at the time of the award (early career researcher = 35 years and younger, mid-career researcher = 36–55 years old, senior researcher = 56 years and older) and calculated median difference scores for each age group. Because the age distributions of the two prizes have limited overlap, we also explored whether there were different patterns in the two awards in the age range where both awards have substantial representation of laureates. This age range was determined to be 47–62 years, where in all included 3-year intervals (ages 47–49, 50–52, 52–55, 55–58, 58–61, 61–64, 64–67) each prize accounts for at least 20% of the total number of scientists in that interval.

## 3. Results

### 3.1. Population characteristics

**Figure 1.**
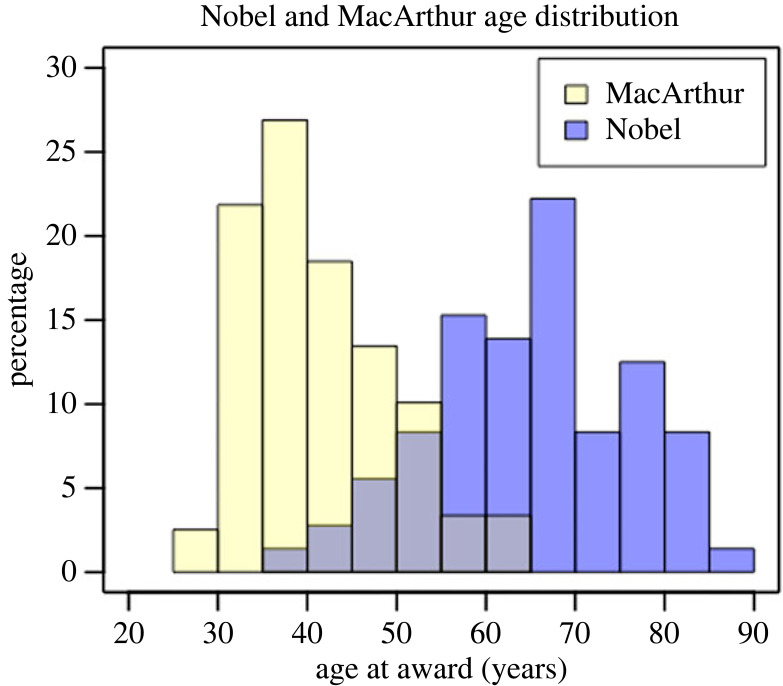
Nobel and MacArthur age distribution.

Nobel Laureates were significantly older (mean 66, s.d. 11) than MacArthur Fellows (mean 42, s.d. 8) (figure 1).

### 3.2. Main results

**Figure A2.**
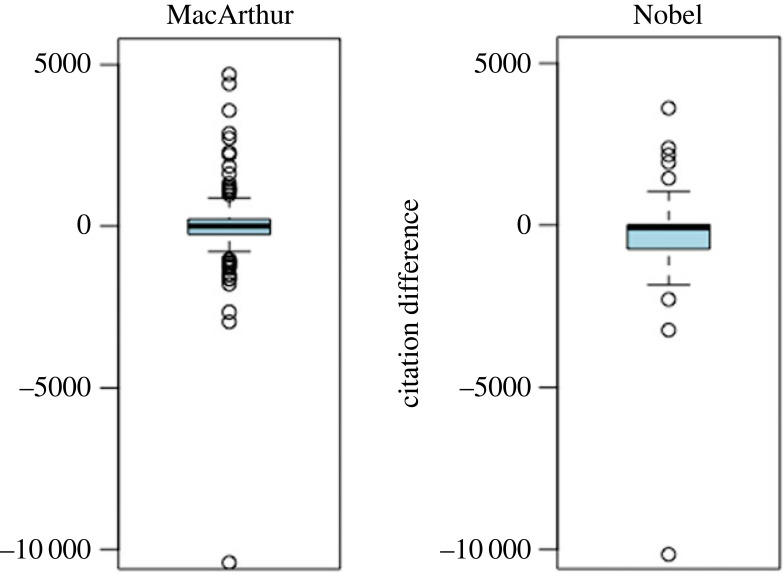
Boxplots of pre- and post-award citation difference scores by award type.

Nobel Laureates and MacArthur Fellows received fewer citations for post-award work than for pre-award work (table 1). The difference was driven predominantly by Nobel Laureates while there was little difference, on average, for pre- versus post-award citation impact for MacArthur Fellows. The median decrease was 80.5 citations among Nobel Laureates and 2 among MacArthur Fellows. For Nobel Laureates, the decrease reached statistical significance (Wilcoxon signed-rank test *p* = 0.004), whereas for MacArthur Fellows the decrease was not statistically significant (Wilcoxon signed-rank test *p* = 0.857). For a boxplot of the difference scores, see Appendix figure A2.

**Table 1.** Median difference in citations (post - pre), publications (post - pre), citations per publication (post - pre) and Wilcoxon signed-rank test of difference scores.

difference metric	group	median	IQR	Wilcoxon signed-rank test	
				effect size (*r*)	*p*-value
citations (post - pre)	Nobel	−80.5	(−733, 5.5)	0.336	0.004
	MacArthur	−2	(−252.5, 212.5)	0.016	0.857
	Pooled	−26	(−379, 168.5)	0.119	0.100
publications (post - pre)	Nobel	−0.5	(−7, 3)	0.158	0.180
	MacArthur	1	(−2, 9.5)	0.293	0.001
	Pooled	0	(−4, 6)	0.119	0.077
citations per publication (post - pre)	Nobel	−6.5	(−22, 7)	0.239	0.043
	MacArthur	−3.3	(−20.8, 5.2)	0.259	0.005
	Pooled	−3.95	(−21.7, 5.4)	0.204	4 × 10^-4^

**Figure 2.**
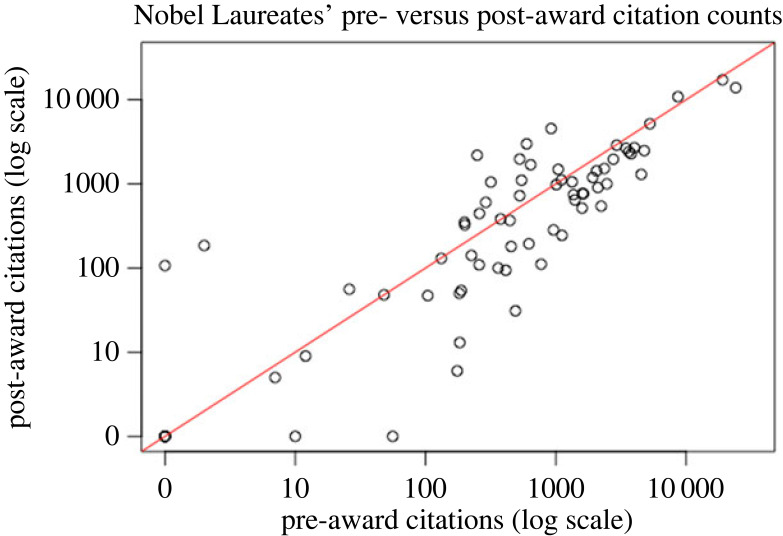
Pre- versus post-award citation counts. Each dot represents one Nobel Laureate. Red line is *y* = *x*.

**Figure 3.**
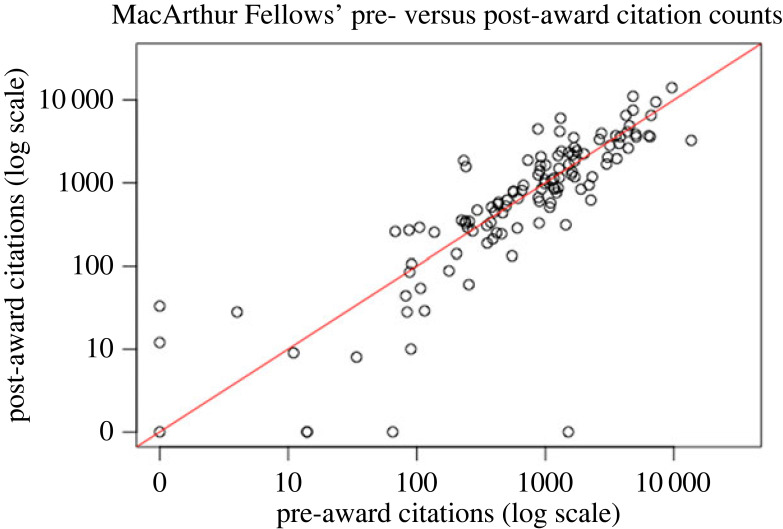
Pre- versus post-award citation counts. Each dot represents one MacArthur Fellow. Red line is *y* = *x*.

Post-award citation impact was lower than the pre-award citation impact for 45 of 72 (62.5%) Nobel Laureates and for 63 of the 119 (52.9%) MacArthur Fellows. Figure 2 shows the scatter-plot of pre-award work citations versus post-award work citations for the Nobel Laureates and figure 3 shows the same scatter-plot for the 119 MacArthur Award Fellows. There was very strong correlation between the pre-award and post-award citation impact for Nobel Laureates (Pearson correlation coefficient *r* = 0.926, *p* < 0.00000001) and strong correlation between the pre-award and post-award citation impact for MacArthur Fellows (*r* = 0.705, *p* < 0.000000001).

### 3.3. Pooled results

Our combined data, including Nobel Laureates and MacArthur Fellows, suggests that awardees tended to earn fewer citations for post-award work than pre-award work (median difference 26 citations, *p* = 0.100).

### 3.4. Publication counts

Nobel Laureates experienced a non-significant decrease (median = −0.5, Wilcoxon signed-rank test *p* = 0.18) in publication counts post-award, and MacArthur Fellows experienced a significant increase (median = 1, Wilcoxon signed-rank test *p* = 0.001) in publication counts post-award work (table 1).

### 3.5. Citations per publication

We observed a significant decrease in post-award versus pre-award citations per publication among Nobel Laureates (median = −6.5, Wilcoxon signed-rank test *p* = 0.043), MacArthur Fellows (median = −3.3, Wilcoxon signed-rank test *p* = 0.005), and in the pooled population (median = −3.95, Wilcoxon signed-rank test *p* = 0.0004) (table 1).

### 3.6. Age-stratified analysis

As shown in table 2, an exploratory analysis according to age at the time of award showed that the declining citations pattern was seen only for researchers who were 42 or older at the time of the award, while an opposite pattern was seen for early career researchers who were given an award (especially MacArthur award) at an age of 41 or younger.

**Table 2.** Median difference in citations (post - pre), publications (post - pre), and citations per publication (post - pre), and interquartile range, stratified by age.

difference metric	age (years)	nobel laureates			MacArthur Fellows			pooled		
		median	IQR	*n*	median	IQR	*n*	median	IQR	*n*
citations (post - pre)	<42	−2074	(−2185, −1963)	2	41	(−257, 466)	66	38	(−337, 399)	68
	42–57	−660	(−1228, 7)	17	−26	(−205, 158)	47	−61	(−477, 154)	64
	>57	−49	(−319, 5)	53	−110	(−273, 2)	6	−56	(−320, 18)	59
publications (post - pre)	<42	18.5	(10.75, 26.25)	2	3	(−1.00, 9.75)	66	3	(−1.00, 10.25)	68
	42–57	−2	(−3, 3)	17	−1	(−5, 7)	47	−1	(−4.00, 3.50)	64
	>57	−1	(−8, 2)	53	2	(−0.5, 13.5)	6	0	(−7.00, 2.50)	59
citations per publication (post - pre)	<42	−108.2	(−126.7, −89.7)	2	−3.2	(−28.0, 6.9)	66	−3.4	(−29.2, 6.8)	68
	42–57	−17.6	(−52.0, 0.0)	17	−4.5	(−14.8, 2.3)	47	−7.7	(−20.7, 2.2)	64
	>57	−2	(−20.1, 13.6)	53	−10.6	(−24.6, −0.2)	6	−3.3	(−20.3, 11.6)	59

Publication counts exhibited similar age trends, with younger researchers tending to publish more papers post-award and older researchers tending to publish fewer.

Across all age strata, researchers tended to accumulate fewer citations per paper after winning their respective research prize.

### 3.7. Age-matched analysis

To investigate heterogeneous treatment effects between different research prizes, we performed a secondary analysis restricted to mid-career researchers, aged 47–62. In this age rage, MacArthur Fellows and Nobel Laureates were more evenly represented than among early-career and senior researchers. Although the median difference scores for citation counts, publication counts and citations per publication were all negative (with the exception of one median difference score of 0), we found limited differences between Nobel Laureates and MacArthur Fellows, and none of the difference scores in any metric in this age stratum reached statistical significance (table 3).

**Table 3.** Median difference in citations, publications, and citations per publication (post - pre) for scientists 47–62 years old (range where both Nobel and MacArthur Laureates are well represented).

difference metric	group	median difference in citations (post - pre)	IQR	Wilcoxon signed-rank test	
				effect size (*r*)	*p*-value
citations (post - pre)	Nobel	−454	(−1206.2, 187.8)	0.258	0.207
	MacArthur	−65	(−130.0, 82.5)	0.123	0.492
	Pooled	−80	(−493.0, 166.5)	0.195	0.148
publications (post - pre)	Nobel	−2	(−4.5, 3.0)	0.180	0.379
	MacArthur	0	(−4.0, 9.5)	0.111	0.537
	Pooled	−1	(−4.0, 3.0)	0.239	0.077
citations per publication (post - pre)	Nobel	−11.9	(−35.8, 23.0)	0.084	0.681
	MacArthur	−6.8	(−15.8, 3.4)	0.330	0.066
	Pooled	−7.1	(−22.0, 8.7)	0.212	0.115

## 4. Discussion

Our analysis expands the evidence that major awards may be associated with decreased impact after receiving the award which has been suggested by previous investigations [1,6,7,9]. To our knowledge, our analysis is the first to investigate how age modifies the effect of winning a research prize on subsequent productivity. Prior analyses investigated the effect of winning a research prize without considering the age at which a recipient wins the prize. Researchers have also previously studied age-creativity dynamics in Nobel Laureates, looking at age at the time of prize-winning contributions rather than age at time of award. [10]. Moreover, our analysis considered a broad sample of mid-career researchers from diverse fields through our sample of MacArthur Fellows. Other studies focus either on an older population (e.g. Nobel Laureates) or on a younger population in specific field (e.g. Fields Medal or John Bates Clark Medal). We also obtained additional insights by juxtaposing analyses of citation counts, publication counts and citations per paper.

Our finding that Nobel Laureates accumulated significantly fewer citations for work completed in years directly after winning the prize than they did in years immediately prior is consistent with findings of previous work [1,6,9]. Conversely, we found no substantial changes in post-award citation profiles of MacArthur Fellows. The different patterns between the two awards are also consistent with prior reports that different awards have varied effects on winners' subsequent research impact ([1,6–8]; and [9]). For both awards, we found strong correlation between the levels of pre- and post-award citation impact across the different awardee scientists, and the correlation was even stronger for Nobel Laureates.

Our combined findings that Nobel Laureates experienced a non-significant post-award decrease in publication counts, MacArthur Fellows experienced a significant post-award increase in publication counts, and all researchers experienced a significant post-award decrease in citations per publication indicates that changes in research impact may be driven by changes in per-paper citations, rather than manuscript publication rates. There are many mechanisms that might be consistent with these patterns, such as researchers branching out to topics studied by fewer researchers who might cite them, higher risk studies, genuine drop in the ability to do influential work, or a winner's curse phenomenon with regression-to-the-mean, in which a scientist who comes up with something major (an extreme value of success) may be unable to reach again such heights (regression-to-the mean) from that extreme value. Causes may differ between individual laureates, and it would be precarious to generalize.

In an exploratory age-stratified analysis, we found that researchers early in their careers may still earn more citations after winning a research award than before. However, very few researchers win awards of this caliber by age 42, and the result for the early career stratum may well have been a chance finding. Although the MacArthur Fellowship and Nobel Prize selection committees share a stated goal of assisting winners in realizing their potential more fully, in terms of citation counts neither the MacArthur Fellowship nor the Nobel Prize heralded increased research impact for the subsequent work and for Nobel Laureates there was even a significant decline.

To investigate between-prize heterogeneity, we also conducted an exploratory award-stratified analysis, in which we restricted to mid-career researchers (ages 47–62), who were better represented among both MacArthur Fellows and Nobel Laureates. We found limited differences between Nobel Laurates and MacArthur Fellows, and none of the difference scores in any metric in this age stratum reached statistical significance, but the data need to be interpreted with caution given the smaller overall sample size in this analysis.

## 5. Limitations

Though we observed a statistically significant effect, and included all scientist awardees to avoid selection bias, we acknowledge that by studying a larger population of honorees drawn from a longer time period we may obtain a more precise effect estimate and enable exploration of sources of heterogeneity to identify specific features of scientists who boost their impact after the award. Moreover, by applying impact criteria other than number of times work was cited (a widely used, but imperfect measure of impact), lengthening or shortening the pre- and post-award windows for citation counts, or making other methodological alterations—including a broader range of awards, for example—we might obtain different results.

We also acknowledge that we cannot probe more complex lines of investigation in the path of the career of a scientist. For example, a scientist who receives more or fewer post-award citations may simply be continuing an existing trend independent of the award, and this trend may be shaped by decisions on what to study and the popularity of the chosen subject matter(s). It is also conceivable that after receiving the most prestigious awards, some scientists may venture into extremely high-risk or unusual topics less likely to be cited. Moreover, recipients of these major prizes have already achieved work of tremendous importance, difficult to match or surpass.

Because this paper uses pre-award citations as controls, if a scientist receives more or fewer citations to her/his pre-award papers as a result of winning an award, our control will be affected. For example, the citation boost phenomenon, where researchers begin citing an author's other papers after s/he publishes a groundbreaking paper [15], has been documented. However, a citation boost resulting from winning a research prize disproportionately increases citation counts of post-award work, since pre-award papers accumulate citations for a few to several years in the absence of such a boost. If anything, the citation boost phenomenon might favour post-award work, strengthening our case that awards do not stimulate productivity.

Overall citation counts in most fields have been increasing over time; a possible influence is the growing size of the scientific workforce and the volume of published papers. Absence of an increase in citation counts in the post- versus pre- period might actually indicate a relative decrease in impact of post-award work, if impact is measured as a share of all citations received in that time frame. While we chose the pre- and post- periods to be only four years apart, a small bias due to growing citations over time is possible. If anything, it strengthens our observation that major awards are often followed by decreased impact.

## 6. Proposed mechanisms

Zuckerman [1,6], Merton [16] and Chan *et al*. [8] proposed that awardees have greater access to resources. With this leverage, an awardee may perform more impactful work. With their newfound status, winners of research prizes may also enjoy a greater chance of success in publishing in more prestigious journals. Other researchers may be more likely to presume that their work is of high quality and thus worth citing. However, as noted also by Zuckerman [1,6] Nobel Laureates decrease the attention they devote to direct research in favour of other activities such as advocating public support for science, mentoring other researchers, writing and speaking to the general public, advocating for policy, and fielding requests from the public. An awardee's post-award works might be less numerous or less impactful per article than her/his pre-award work because s/he devotes more attention to such matters. Post-award increase in citations of the youngest MacArthur fellows may reflect a lower level of engagement in these activities.

Zuckerman [1,6], and Li *et al*. [9] documented that Nobel Laureates more often venture into new fields of research after receiving their prize. A prize-winning researcher might transition to a field with more or fewer average citations per paper.

Perhaps more importantly, one may need to consider the phenomenon of ‘one hit wonders’ and regression to the mean. The publication(s) for which a scientist wins a research prize may represent the pinnacle of her/his contribution, after which s/he regresses towards a baseline level of impact. This theory will bear on the results in our analysis only if a researcher's prize-winning papers were published during the pre-award citation count window. This is almost never the case for Nobel Laureates (awards usually are given several decades after a major discovery), but may affect some of the MacArthur Fellows. Fortunato [17] observed that the number of years between when a scientist makes a discovery for which they later receive a Nobel prize and their receipt of that prize has increased substantially, with intervals of over 20 years becoming common.

Finally, Zuckerman [1,6] and Li *et al*. [9] observed that awardees are more likely to terminate prior collaborations and develop new ones after winning. In some cases, this may occur because recent awardees are also more likely to switch fields, which may entail working with scientists already established in those fields. Zuckerman also proposes that disruption of collaborations may be partly explained by changes in recent awardees' professional relationships resulting from their sudden increase in status.

## 7. Future directions

Use of awards in scientific investigation is one topic in a broad discussion of rewards and incentives that includes money (e.g. salaries, grants), promotion, tenure, and various accolades and recognitions. People are increasingly recognizing that much of the reward system in science is without basis in evidence, and that reform incorporating criteria and reward practices better aligned with high-quality, reproducible, rigorous work is essential to reap the full benefits of science [18,19].

Major awards represent an extreme in the continuum of rewards, and they directly affect very few people, but they may indirectly shape the attitudes and actions of large numbers of scientists. In this regard, they are potentially powerful tools for good—or harm. Studying their impact is challenging, because experimental methods, e.g. randomization, may seem impossible. However, award-granting institutions might compile lists of scientists of similar caliber and randomly assign some of them to win an award. For major awards, the list of deserving individuals is usually very long. Even if the winner is chosen without randomization, one might compare trajectories of winners versus nominees. Nobel lists are released after 50 years so such analyses are possible, though long delay makes inferences less generalizable to the present. For other major awards, analyses of more recent recipients and runners-up may offer useful insights.

While we and others have directed attention to the effect of a research prize on a recipient, awards likely motivate other scientists to work in ways or on topics they perceive will increase their chances of someday being honored. Characterizing the effect of research awards on scientists other than awardees may merit future study, as well. Moreover, although the awards that we evaluated are personal awards, much of current science is collaborative. Experimenting with team-level awards and comparing their impact on the scientific ecosystem with that of personal awards may also yield insight into how to motivate scientists to contribute more to common good.

## Data Availability

Public link to code and datasets: https://doi.org/10.5061/dryad.pc866t1rx [14]. Please note that the link grants access to data in the form of .xlsx and .csv files. Analysis scripts are in the form of R and python scripts and must be manually downloaded from the reviewer link's landing page under text displaying ‘Software files available at Zenodo.’

## Appendix

**Inclusion of MacArthur Fellows based on field of study**

The MacArthur Foundation labels awardees' work with one or more of 91 fields, called ‘areas of focus’ on their website. For example, the MacArthur Foundation labels Fellow Angela Belcher's work with one field—Materials Science and Engineering—and Fellow John Ochsendorf's work with three fields—Civil and Environmental Engineering, Art History/Theory/Criticism and Visual Culture, Culture and Society.

The MacArthur Foundation classifies each field into one of five larger categories: The Arts (18 fields); Humanities (16 fields); Public Issues (22 fields); Science, Technology, Engineering, and Mathematics (24 fields); and Social Science (11 fields). Of 91 fields on the MacArthur Foundation website, 90 are unique; Biological Anthropology is listed under Science, Technology, Engineering, and Mathematics and under Social Science. Appendix table A1 below lists all fields enumerated on the MacArthur website.

Our sample of 238 individuals who won a MacArthur Fellowship from 2004–2013 represented 67 of 90 unique fields, 15 of 18 Arts fields, 8 of 17 Humanities fields, 20 of 22 Public Issues fields, 22 of 24 STEM fields, and 6 of 11 social science fields.

111 Fellows worked in Science, Technology, Engineering, and Mathematics + Social Science fields, 89 Fellows worked in Arts and Humanities only, and 38 were in Public Issues or Public Issues + Arts and Humanities. We included Fellows based on their field of study if the MacArthur Foundation applied any STEM or Social Science label to their work, and we excluded Fellows if they had only Arts or Humanities labels. The remaining fellows had public issues labels but no STEM labels. We reviewed these fellows' publication histories in greater detail to determine their eligibility for analysis.

We excluded 89 Fellows for working only in field(s) that the MacArthur Foundation website labeled as Arts and Humanities, and 30 of the remaining 149 STEM and/or Public Issues Fellows for having fewer than 100 total career citations in Scopus, including one individual who had no Scopus profile. In summary, 119 Fellows met inclusion criteria, and 119 did not. See Appendix figure A1 below for a visual representation of inclusion criteria.

**Table A1.** List of MacArthur Fellowship fields.

Arts	
Architecture and Environmental Design	Moving Image
Arts Education	Music Performance and Composition
Arts Entrepreneurship/Management	Performance Art
Comics and Graphic Narratives	Photography
Community/Social Practice	Poetry
Crafts and Arts Technology	Theatrical Arts
Curation, Collecting, and Conservation	3-D Visual Art
Choreography and Dance	2-D Visual Art
Graphic Design and Illustration	Fiction and Nonfiction Writing
Humanities	
African History	Latin/South American History and Mesoamerican History
American History	Literary History and Criticism
Art History/Theory/Criticism and Visual Culture	Middle Eastern History
Asian History	Musicology and Ethnomusicology
Classics, Late Antiquity, and Medieval Studies	Philology
Cultural/Intellectual History and History of Religion	Philosophy
Curation, Collecting, and Conservation	History of Science and Technology
Environmental History	Translation
Early Modern and Modern European History	—
Public Issues	
Ageing	Food and Agriculture
Health Care Delivery	Housing and Community/Economic Development
Children and Youth Services	Health Policy
Civil Rights and Civil Liberties	Human Rights and Human Security
Conservation	Immigration
Criminal Justice	Communications and Journalism
Culture and Society	Low-Income Individuals/Families and the Disadvantaged
Persons with Disabilities	Peace and International Security
Education and Training	Privacy and Intellectual Property
Energy and Natural Resource Policy	Civil Society and Community Organizing
Environment and Climate Change	Invention and Adaptive Technology
Science, Technology, Engineering, and Mathematics	
Aerospace Engineering	Genetics and Molecular Biology
Astrophysics and Astronomy	Public Health and Biomedical/Health Sciences
Biological Anthropology	Materials Science and Engineering
Biochemistry, Biophysics, and Structural Biology	Mathematics, Statistics, and Probability
Bioengineering and Biotechnology	Mechanical Engineering
Cell, Developmental, and Systems Biology	Microbiology, Virology, and Immunology
Chemical Engineering	Neuroscience and Neurobiology
Chemistry	Oceanography, Atmospheric Sciences, and Meteorology
Civil and Environmental Engineering	Paleontology
Computer Science and Electrical Engineering	Physics
Earth Sciences	Plant Sciences and Forestry/Forest Science
Ecology and Evolutionary/Environmental Biology	STEM Education and Communication
Archaeology	Linguistics
Biological Anthropology	Media Studies
Cultural Anthropology	Political Science
Economics	Psychology and Cognitive Science
Geography	Sociology
Legal Studies	—

As an additional quality control measure, and to account for factors other than those stated in the inclusion criteria AN and HB independently reviewed MacArthur Fellows’ profiles on the MacArthur website.

**Citation boxplots**
